# Hydrated Sodium Ion Clusters [Na^+^(H_2_O)_n_ (*n* = 1–6)]: An *ab initio* Study on Structures and Non-covalent Interaction

**DOI:** 10.3389/fchem.2019.00624

**Published:** 2019-09-12

**Authors:** Pengju Wang, Ruili Shi, Yan Su, Lingli Tang, Xiaoming Huang, Jijun Zhao

**Affiliations:** ^1^Key Laboratory of Materials Modification by Laser, Ion and Electron Beams (Dalian University of Technology), Ministry of Education, Dalian, China; ^2^School of Mathematics and Physics, Hebei University of Engineering, Handan, China; ^3^College of Science, Dalian Nationalities University, Dalian, China; ^4^School of Ocean Science and Technology, Dalian University of Technology, Panjin, China

**Keywords:** hydrated sodium cluster, stabilization energy, anharmonic effect, IR spectra, natural bond orbital

## Abstract

Structural, thermodynamic, and vibrational characteristics of water clusters up to six water molecules incorporating a single sodium ion [Na^+^(H_2_O)_n_ (*n* = 1–6)] are calculated using a comprehensive genetic algorithm combined with density functional theory on global search, followed by high-level *ab initio* calculation. For *n* ≥ 4, the coordinated water molecules number for the global minimum of clusters is 4 and the outer water molecules connecting with coordinated water molecules by hydrogen bonds. The charge analysis reveals the electron transfer between sodium ions and water molecules, providing an insight into the variations of properties of O–H bonds in clusters. Moreover, the simulated infrared (IR) spectra with anharmonic correction are in good agreement with the experimental results. The O–H stretching vibration frequencies show redshifts comparing with a free water molecule, which is attributed to the non-covalent interactions, including the ion–water interaction, and hydrogen bonds. Our results exhibit the comprehensive geometries, energies, charge, and anharmonic vibrational properties of Na^+^(H_2_O)_n_ (*n* = 1–6), and reveal a deeper insight of non-covalent interactions.

## Introduction

Hydrated ion clusters widely exist in oceans and living organisms, especially hydrated sodium ion clusters, which are important in the control of blood pressure, cell permeability, neuronal activity, and other somatic functions (Jensen, 1992; Feller et al., 1994; Pohl et al., 2013). Understanding the behavior of hydrated sodium ion clusters is helpful to uncover the mechanism of some key biochemical reactions (Mano and Driscoll, 1999; Snyder, 2002; Dudev and Lim, 2010; Payandeh et al., 2011). A number of experimental (Dzidic and Kebarle, 1970; Tang and Castleman, 1972; Schulz et al., 1986, 1988; Blades et al., 1990; Hertel et al., 1991; Patwari and Lisy, 2003; Vaden et al., 2004; Mancinelli et al., 2007) and theoretical (Perez et al., 1983; Arbman et al., 1985; Lybrand and Kollman, 1985; Cieplak et al., 1987; Probst, 1987; Bauschlicher et al., 1991; Dang et al., 1991; Perera and Berkowitz, 1991; Hashimoto and Morokuma, 1994; Glendening and Feller, 1995; Kim et al., 1995; Ramaniah et al., 1998; Carrillo-Tripp et al., 2003; Lee et al., 2004; Rao et al., 2008; Neela et al., 2012; Biring et al., 2013; Dinh et al., 2014; Soniat et al., 2015; Fifen and Agmon, 2016) studies on hydrated sodium ion clusters have been reported, particularly on the global minima. A global minimum can be determined by obtaining the stabilization energies of isomers. In experiments, comparing enthalpies is the most direct method to obtain thermodynamic information to deduce stabilization energies. With a high-pressure mass spectrometer containing a thermionic alkali ion source, Dzidic and Kebarle reported the enthalpies and entropies of hydrated sodium ion clusters for *n* = 1–6 in gas phase (Dzidic and Kebarle, 1970). With the hydration number increasing, the binding energy per water molecule decreases. Glendening and Feller calculated the stabilization energies and stabilization enthalpies of Na^+^(H_2_O)_n_ (*n* = 1–6) at various levels of theory (Glendening and Feller, 1995), in which the RHF and MP2 levels with the 6-31+G^*^ basis set reproduced the experimental values obtained by Dzidic and Kebarle well (Dzidic and Kebarle, 1970).

For the structures of hydrated sodium ion clusters, the coordination number is of particular appeal to studies in different phases. In liquid water, the coordination number of a sodium ion is about 5.5 ± 0.5 based on molecular dynamics simulation (Mancinelli et al., 2007; Megyes et al., 2008; Bankura et al., 2013, 2014; Lev et al., 2013; Galib et al., 2017; Liu et al., 2019). However, in gas-phase clusters, the coordination number is 4 from *ab initio* calculations at 0 K (Kim et al., 1995; Neela et al., 2012; Soniat et al., 2015; Fifen and Agmon, 2016). The structures of Na^+^(H_2_O)_n_ (*n* = 1–4), all the water molecules surrounding the sodium ion, were firstly reported by Bauschlicher et al. from *ab initio* calculation (Bauschlicher et al., 1991). For Na^+^(H_2_O)_5_, 4+1+0 (the structures of isomers are presented in the form of *n*_1_+*n*_2_+*n*_3_, where *n*_1_, *n*_2_, and *n*_3_ are the numbers of water molecules in the first, second, and third solvation shells, respectively) is supported as the global minimum by most *ab initio* calculations at 0 K (Hashimoto and Morokuma, 1994; Kim et al., 1995; Lee et al., 2004; Rao et al., 2008; Neela et al., 2012; Biring et al., 2013; Soniat et al., 2015; Fifen and Agmon, 2016), and 5+0+0 is deemed to be concomitant with 4+1+0 at 298 K (Kim et al., 1995; Fifen and Agmon, 2016). For *n* = 6, at 0 K, several recent *ab initio* calculations stated that 4+2+0 (with *D*_2*d*_ symmertry) is the global minimum (Lee et al., 2004; Rao et al., 2008; Biring et al., 2013; Soniat et al., 2015; Fifen and Agmon, 2016), which was proposed by Lybrand and Kollman (1985) based on RWK2 potential (Reimers et al., 1982). However, Neela et al. sustained 5+1+0 to be the global minimum for *n* = 6 calculated at MP2/cc-pVTZ level of theory (Neela et al., 2012). At room temperature, Kim et al. found that 5+1+0 possesses better stability than 4+2+0 by HF/TZ2P (Kim et al., 1995). Differently, Fifen and Agmon indicated that 4+1+1 is dominant, calculated at MP2/6-31++G(d,p) (Fifen and Agmon, 2016).

The infrared (IR) spectra are available in distinguishing the cluster isomers. For hydrated sodium ion clusters, the feature peaks of O–H stretching mode could accurately provide the structure information of clusters (Huang and Miller, 1989; Patwari and Lisy, 2003; Vaden et al., 2004; Miller and Lisy, 2008a,b; Ke et al., 2015), in which the non-covalent interactions, including ion–water interaction and hydrogen bond, weaken the O–H bonds, causing redshifts for O–H stretching vibration modes and producing different feature peaks for different structures (Muller-Dethlefs and Hobza, 2000; Vaden et al., 2002, 2004, 2006; Kozmutza et al., 2003; Bush et al., 2008; Miller and Lisy, 2008a), Using a custom-built, triple-quadrupole mass spectrometer as well as *ab initio* calculations, Lisy et al. reported the IR spectra of Na^+^(H_2_O)_n_ (*n* = 2–5) and Na^+^(H_2_O)_n_Ar (*n* = 2–5) (Miller and Lisy, 2008a,b; Ke et al., 2015). For *n* = 4, 3+1+0 is the stable structure with bent hydrogen bonds (Miller and Lisy, 2008a). Recently, through straightforward IR spectra for *n* = 5, they speculated that 4+1+0 and 3+1+1 could be concomitant at 75 K (Ke et al., 2015).

In spite of many works having been conducted for Na^+^(H_2_O)_n_ (*n* = 1–6), the global minima of *n* = 4–6 remains unclear. Moreover, the non-covalent interaction and electron transfer in hydrated sodium ion clusters have not been discussed in detail, which can elucidate the principle of the shifts of O–H stretching frequency. In this paper, the comprehensive genetic algorithm combined MP2 method is used to determine the global minima of Na^+^(H_2_O)_n_ (*n* = 1–6) and simulate their anharmonic vibrational frequencies. Furthermore, charge transfer inside the clusters through NBO analysis and charge density difference are contained, aiming to reveal the principle of non-covalent interactions effecting on the O–H bonds.

## Methods

In this work, some of the structures of the Na^+^(H_2_O)_n_ (*n* = 1–6) clusters are adopted from previous literatures (Bauschlicher et al., 1991; Glendening and Feller, 1995; Ke et al., 2015; Fifen and Agmon, 2016). To obtain more isomers for *n* = 4–6, a global search with the comprehensive genetic algorithm (CGA, Zhao et al., 2016) combined with DMol^3^ program (Delley, 2000) based on DFT was executed. The CGA method is described in our previous review in detail (Zhao et al., 2016). For each cluster size with *n* ≥ 4, we took 10 independent global searches, and for each search, we maintained mating and mutation operations on a population of eight members of up to 3000 GA iterations. Since the Becke-Lee-Yang-Parr (BLYP, Becke, 1988; Lee et al., 1988) functional would provide similar relative energies to MP2 (Møller and Plesset, 1934) method (see Table S1), the generalized gradient approximation (GGA) with the BLYP functional and *p-* and *d-* polarization functions (DNP) basis sets were employed to optimize the clusters' isomers in CGA search without symmetry constraint. Considering the calculation cost, zero-point energy (ZPE) was not contained in global search.

Our previous work proved that MP2 is a reasonable method to obtain the energies and properties of small hydrogen-bonded systems (Liu et al., 2013; Shi et al., 2017, 2018). Since the geometrical optimization at augmented correlation-consistent polarized valence double-zeta (aug-cc-pVDZ, Dunning, 1989; Kendall et al., 1992) and aug-cc-pVTZ provide almost the same structures (see Table S2), MP2/aug-cc-pVDZ method was utilized to optimize the isomer structures. Single-point energies of these clusters were computed at MP2/aug-cc-pVQZ and MP2/aug-cc-pVDZ levels.

Within harmonic approximation, MP2 calculation usually overestimates the frequencies relative to the experiment, especially for the high frequencies in IR spectra, and may leave out some peaks. Hence, we calculated the IR spectra with anharmonic correction at MP2/aug-cc-pVDZ level at 298 K *via* second-order vibrational perturbation theory (VPT2, Barone, 2005; Barone et al., 2010), as well as to obtain ZPE and thermal correction at 298 K.

For visualizing the bonding strength between the two atoms intuitively, NBO (Carpenter and Weinhold, 1988; Reed et al., 1988) was calculated at MP2/aug-cc-pVQZ level, as well as to obtain the Wiberg bond order (Wiberg, 1968). All the calculations aforementioned were performed in the Gaussian 09 package (Frisch et al., 2013).

Charge density differences of 1+0+0, 2+0+0, 3+0+0, 3+1+0, 4+0+0, and 4+1+0 were calculated using GGA and Perdew–Burke–Ernzerhof (PBE, Perdew et al., 1996) functional, the projector-augmented wave potentials (Blochl, 1994) with an energy cutoff of 500 eV, as implemented in Vienna Ab-initio Simulation Package (VASP, Kresse and Furthmuller, 1996). Only Γ point is k-point with a vacuum layer of over 15 Å was employed in our calculation. The charge density difference is given by:

(1) $$\begin{array}{r} \Delta \rho =\rho_{cluster}-\rho_{Na^{+}}-\rho_{H_{2}O} \end{array}$$

where ρ_*cluster*_, $\rho_{Na^{+}}$, and ρ_*H*_2_*O*_ are the charge density of entire hydrate cluster, sodium ion and all the water molecules, respectively.

## Results and Discussion Structures

**Figure 1:**
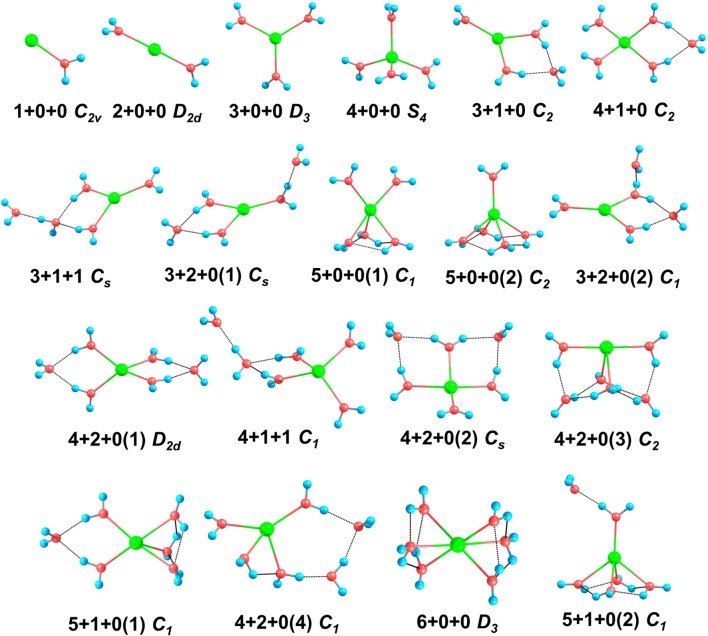
The structures and symmetries of Na^+^(H_2_O)_n_ (*n* = 1–6) optimized at MP2/aug-cc-pVDZ level of theory. Blue, red, and green balls denote hydrogen, oxygen, and sodium atoms, respectively. The black dashed lines represent hydrogen bonds.

We re-optimized all the isomer clusters obtained from CGA search using the MP2/aug-cc-pVDZ method. The optimized structures and symmetries of Na^+^(H_2_O)_n_ (*n* = 1–6) are present in Figure 1. Due to the high computational cost, we used the total energies computed at the MP2/aug-cc-pVQZ//MP2/aug-cc-pVDZ+ZPE level to rank the energy order of all the isomers. Table 1 lists the relative energies at 0 K and 298 K calculated at MP2/aug-cc-pVQZ, MP2/aug-cc-pVDZ, and BLYP/DNP levels, respectively.

**Table 1:** Relative energies (in units of kcal/mol) of Na^+^(H_2_O)_n_ (*n* = 1–6) at 0 K and 298 K calculated at MP2/aug-cc-pVQZ, MP2/aug-cc-pVDZ and BLYP/DNP levels, respectively.

	**MP2/aug-cc-pVQZ**		**MP2/aug-cc-pVDZ**		**BLYP/DNP**	
	**0 K**	**298 K**	**0 K**	**298 K**	**0 K**	**298 K**
1+0+0	0	0	0	0	0	0
2+0+0	0	0	0	0	0	0
3+0+0	0	0	0	0	0	0
4+0+0	0	0	0	0	0	0
3+1+0	1.36	0.04	3.30	1.98	3.32	2.00
4+1+0	0	0	0	0	0	0
3+1+1	2.00	1.48	3.61	3.09	2.68	2.16
3+2+0(1)	2.09	1.50	3.18	2.59	2.47	1.88
5+0+0(1)	2.77	2.51	2.53	2.28	4.16	3.90
5+0+0(2)	2.99	2.41	3.52	2.94	4.81	4.22
3+2+0(2)	2.99	2.68	3.95	3.64	3.55	3.24
4+2+0(1)	0	0	0	0	0	0
4+1+1	1.05	1.72	0.73	1.41	0.14	0.81
4+2+0(2)	1.25	1.40	1.15	1.31	1.63	1.79
4+2+0(3)	2.22	0.76	4.58	3.12	4.48	3.02
5+1+0(1)	2.30	2.15	2.22	2.08	3.76	3.61
4+2+0(4)	3.45	3.28	3.57	3.40	3.37	3.20
6+0+0	4.19	3.92	3.93	3.67	6.56	6.30
5+1+0(2)	4.32	4.66	4.45	4.79	5.10	5.43

For *n* = 1–3, the global minima, i.e., 1+0+0, 2+0+0, and 3+0+0, all the water molecules surround the sodium ions and locate at equivalent positions without hydrogen bonds, which are similar to those in previous reports (Hashimoto and Morokuma, 1994; Kim et al., 1995; Rao et al., 2008; Neela et al., 2012; Soniat et al., 2015; Fifen and Agmon, 2016).

For *n* = 4, CGA has located 3+1+0 and 4+0+0 isomers. Among them, 3+1+0 was the better result in all ten CGA global searches, which possesses lower energy than 4+0+0 without ZPE correction (see Table S1). In most previous reports by *ab initio* calculations, 4+0+0 was argued to be the most stable of the structures (Hashimoto and Morokuma, 1994; Kim et al., 1995; Ramaniah et al., 1998; Lee et al., 2004; Rao et al., 2008; Kamarchik et al., 2011; Neela et al., 2012; Fifen and Agmon, 2016), whereas Miller and Lisy reported that the IR spectrum of Na^+^(H_2_O)_4_ is similar to that of 3+1+0 at 300 K (Miller and Lisy, 2008a). From Table 1, at 0 K, our MP2 calculation manifests that 4+0+0 is lower in energy by 1.36 kcal/mol, while 4+0+0 and 3+1+0 possess almost equal energies at 298 K. As shown in Figure 1, 4+0+0 has four equivalent water molecules surrounding the sodium ion, while 3+1+0 is evolved by 3+0+0 connecting a water molecule with two coordinated water molecules by hydrogen bonds.

For *n* = 5, six isomers are contained in our calculation: 4+1+0, 3+1+1, two 3+2+0 structures, and two 5+0+0 structures with different symmetries. 4+1+0 was the best structure in all ten CGA searches, and had the lowest energy at 0 K. 3+1+1, 3+2+0(1), and 5+0+0(1) possess the relative energies of 2.00, 2.09, and 2.77 kcal/mol, respectively. Besides, 3+2+0(2) and 5+0+0(2) have equal relative energies of 2.99 kcal/mol. At 298 K, 5+0+0(2) possesses a lower energy than 5+0+0(1), and the energetic order of the other isomers doesn't change. At the BLYP/DNP level of theory, 3+2+0(1) becomes the second lowest energy structure, and 3+2+0(2) has lower energy than 5+0+0(1) and 5+0+0(2) at 0 K. In Figure 1, it is noteworthy that the global minimum structure 4+1+0 has an extra water molecule located at outer shell of 4+0+0. 3+1+1 structure has a water molecule connecting with the outer water molecule in 3+1+0 *via* a hydrogen bond. Similarly, 3+2+0(1), with *C*_*s*_ symmetry, has a water molecule connecting the isolated coordinated water molecule in 3+1+0, and 3+2+0(2) has a water molecule located beside a coordinated water molecule with a hydrogen bond of 3+1+0. 5+0+0(1) structure has three water molecules form a water cycle via hydrogen bonds, and the other two coordinated water molecules are isolated opposite the water cycle. Differently, 5+0+0(2) has only one isolated coordinated water molecule, with the other four water molecules constituting a quaternary water cycle.

For *n* = 6, we found eight isomers, 4+1+1, 6+0+0, two 5+1+0 structures, and four 4+2+0 structures with different symmetries: *D*_2*d*_, *C*_*s*_, *C*_2_, and *C*_1_. In all ten CGA searches, 4+2+0(3) had the best solution with the lowest energy at MP2/aug-cc-pVQZ without ZPE (see Table S1). From Table 1, at 0 K, 4+2+0(1) has the lowest energy, while the relative energies of 4+1+1 and 4+2+0(2) are 1.05 and 1.25 kcal/mol, respectively. The other five isomers possess relative energies of over 2 kcal/mol. At 298 K, 4+2+0(3) becomes the second lowest energy structure rather than 4+1+1, with the relative energy of only 0.76 kcal/mol. Four isomers with four coordinated water molecules have lower stabilization energies both at 0 K and 298 K, indicating that four coordination is more favorable for *n* = 6. Compared to MP2/aug-cc-pVQZ, the calculations at BLYP/DNP shows that 4+2+0(4) has lower energy than 4+2+0(3) and 5+1+0(1). Besides, 6+0+0 possesses the highest relative energy of 6.56 kcal/mol, which is obviously higher than the total energy interval at MP2/aug-cc-pVQZ (4.32 kcal/mol). Combining with the relative energies of *n* = 5, BLYP/DNP gives the same global minima and similar energetic order to MP2/aug-cc-pVQZ. However, BLYP/DNP would overestimate the energies of the 5 and 6 coordinated structures, indicating that the CGA search tends to provide the isomers with 3 and 4 coordinated water molecules. Since the previous *ab initio* calculations show that the coordination number is 4 at 0 K (Kim et al., 1995; Neela et al., 2012; Soniat et al., 2015; Fifen and Agmon, 2016), the CGA search at BLYP/DNP could provide the global minima and other reliable isomers. As seen in Figure 1, 4+2+0(1) is a water molecule via hydrogen bonds connecting with the two coordinated water molecules in 4+1+0. Three coordinated water molecules in 4+2+0(2) connect the two outer water molecules *via* hydrogen bonds, and the oxygen atoms in these five water molecules locate in a flat with the sodium ion approximatively. 4+2+0(3) with *C*_2_ symmetry forms a water cycle *via* hydrogen bonds between two coordinated water molecules and the two outer water molecules. Besides, 4+2+0(4) with lowest symmetry has a coordinated water molecule without hydrogen bond. The 4+1+1 structure is a water molecule connecting with the outer water molecule in 4+1+0 *via* a hydrogen bond. In addition, 5+1+0(1) is an extra water molecule located at the outer shell of 5+0+0(1), connecting with the two isolated coordinated water molecules. 5+1+0(2) is just a water molecule connecting with the isolated coordinated water molecule in 5+0+0(2) *via* a hydrogen bond. 6+0+0 could transform from the perfect *S*_6_ symmetry to *D*_3_ symmetry, with two water cycles on two sides of the sodium ion, in accordance with previous calculations based on the polarizable electropole model (Perez et al., 1983).

The bond lengths present interesting variation trends as summarized in Table 2. The $\bar{r}$*(Na–O)* increases strictly with the increasing of coordination number, indicating the decreasing of average ion–water interaction. For the structures with two water shells, if a coordinated water molecule is the proton-donor in a hydrogen bond system, the *r(Na–O)* should be shorter. In contrast, if the oxygen atom forms a hydrogen bond, the *r(Na–O)* should become longer. For the *r(O*–*H)*s, each average *r(O*–*H)* of water molecules in clusters is longer than the *r(O*–*H)* of free water molecules (0.966 Å), which stems from the non-covalent ion–water interaction. Meanwhile, the hydrogen bonds also stretch the O–H bonds and make the water molecules asymmetric.

**Table 2:** Average distances between sodium ions and oxygen atoms [$\bar{r}$*(Na–O)*], distances between sodium ions and each oxygen atoms [*r(Na–O)*] and O–H bond lengths [*r(O–H)*] of coordinated water molecules in Na^+^(H_2_O)_n_ (*n* = 1–6) optimized at MP2/aug-cc-pVDZ level of theory.

	**Symmetry**	**$\bar{r}$*(Na–O)*/Å**	***r(Na–O)*/Å**	***r(O–H)*/Å**
H_2_O	*C_2*v*_*			0.966
1+0+0	*C_2*v*_*	2.275	2.275	0.968
2+0+0	*D_2*d*_*	2.302	2.302^(2)^	0.968
3+0+0	*D_3_*	2.335	2.335^(3)^	0.968
3+1+0	*C_2_*	2.322	2.344	0.968
			2.311^(2)^	0.965 0.975
3+1+1	*C_*s*_*	2.316	2.347	0.967
			2.300^(2)^	0.965 0.979
3+2+0(1)	*C_*s*_*	2.312	2.287	0.956 0.982
			2.234^(2)^	0.956 0.974
3+2+0(2)	*C_1_*	2.313	2.351	0.967
			2.314	0.965 0.976
			2.273	0.970 0.978
4+0+0	*S_4_*	2.370	2.370^(4)^	0.967
4+1+0	*C_2_*	2.364	2.378^(2)^	0.967
			2.350^(2)^	0.966 0.974
4+1+1	*C_1_*	2.361	2.384^(2)^	0.967
			2.338^(2)^	0.965 0.978
4+2+0(1)	*D_2*d*_*	2.359	2.359^(4)^	0.965 0.974
4+2+0(2)	*C_*s*_*	2.359	2.379	0.967
			2.361^(2)^	0.965 0.975
			2.337	0.970
4+2+0(3)	*C_2_*	2.411	2.317^(2)^	0.965 0.974
			2.505^(2)^	0.967 0.981
4+2+0(4)	*C_1_*	2.391	2.335	0.968
			2.416	0.966 0.972
			2.339	0.966 0.976
			2.392	0.966 0.984
5+0+0(1)	*C_1_*	2.436	2.466^(3)^	0.967 0.970
			2.408	0.967 0.976
			2.371	0.967
5+0+0(2)	*C_2_*	2.460	2.486^(4)^	0.967 0.972
			2.355	0.967
5+1+0(1)	*C_1_*	2.439	2.491^(3)^	0.967 0.971
			2.360^(2)^	0.965 0.974
5+1+0(2)	*C_1_*	2.472	2.516^(4)^	0.967 0.973
			2.296	0.965 0.981
6+0+0	*D_3_*	2.485	2.485^(6)^	0.967 0.971

*The numbers in the parentheses in the line of r(Na–O)/Å represent that there are equivalent water molecules in this number. The two bond lengths in the line r(O–H)/Å represent that hydrogen bond stretches one O–H bond in this water molecule*.

## Charge Analysis

**Figure 2:**
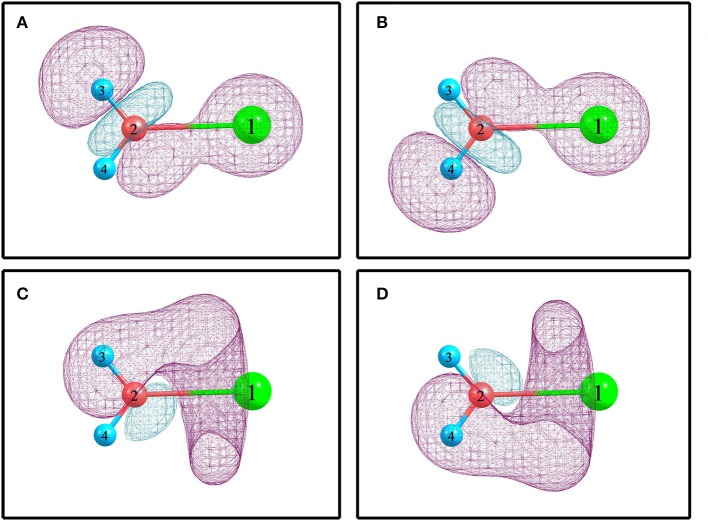
The NBO overlapping and electron transfer in 1+0+0 calculated at the MP2/aug-cc-pVQZ level of theory. **(A)** ${\text{Na}}_{1}^{+}$(s) → σ^*^(O_2_-H_3_). **(B)** ${\text{Na}}_{1}^{+}$(s) → σ^*^(O_2_-H_4_). **(C)** σ(O_2_-H_3_) → ${\text{Na}}_{1}^{+}$(sp^0.66^)^*^. **(D)** σ(O_2_-H_4_) → ${\text{Na}}_{1}^{+}$(sp^0.66^)^*^.

For elucidating the non-covalent interactions in hydrated sodium ion clusters, Figure 2 and Table 3 show the NBO overlapping 3D schematic diagrams and electron transfers of 1+0+0. Figures 2A,B depict the 2s orbital of sodium ion overlaps the O–H anti-bonding orbitals of water molecule, resulting in electron transfer from the sodium ion to the water molecule. In contrast, Figures 2C,D depict the O–H bonding orbitals overlap to the empty orbital of sodium ion, resulting in electron transfer from the water molecule to the sodium ion. From Table 3, the amplitude of *E*^(2)^ manifests that electron transfer from water molecules, including the electrons in O–H bonding orbitals and the oxygen atom's lone pair electron orbitals, to sodium ions is larger than that from sodium ions to water molecules, which synergistically weakens and stretches the O–H bonds of 1+0+0.

**Table 3:** The electron transfer and second-order perturbation energies (*E*^(2)^, expressing the electron delocalization and the extent of charge transfer between different orbitals, in units of kcal/mol) between different natural bond orbitals of 1+0+0 calculated at MP2/aug-cc-pVQZ level of theory.

**Donor orbital**	**Acceptor orbital**	***E^**(2)**^***
${\text{Na}}_{1}^{+}$(s)	σ^*^(O_2_-H_3_)	0.09
${\text{Na}}_{1}^{+}$(s)	σ^*^(O_2_-H_4_)	0.09
${\text{Na}}_{1}^{+}$(p)	σ^*^(O_2_-H_3_)	0.05
${\text{Na}}_{1}^{+}$(p)	σ^*^(O_2_-H_4_)	0.05
σ(O_2_-H_3_)	${\text{Na}}_{1}^{+}$(sp^0.66^)^*^	0.45
σ(O_2_-H_3_)	${\text{Na}}_{1}^{+}$(p)^*^	0.15
σ(O_2_-H_3_)	${\text{Na}}_{1}^{+}$(sp^1.55^d^3.85^f^2.27^g^1.41^)^*^	0.12
σ(O_2_-H_4_)	${\text{Na}}_{1}^{+}$(sp^0.66^)^*^	0.45
σ(O_2_-H_4_)	${\text{Na}}_{1}^{+}$(p)^*^	0.15
σ(O_2_-H_4_)	${\text{Na}}_{1}^{+}$(sp^1.55^d^3.85^f^2.27^g^1.41^)^*^	0.12
O_2_(s)	${\text{Na}}_{1}^{+}$(sp^0.66^)^*^	0.27
O_2_(p)	${\text{Na}}_{1}^{+}$(pd^0.35^f^0.25^g^0.44^)	0.20
O_2_(sp^1.02^)	${\text{Na}}_{1}^{+}$(sp^0.66^)^*^	1.90

The ^*^ in sodium ion orbitals represent that the orbitals are empty. The subscripts of the atoms correspond to the serial numbers in Figure 2.

For revealing the strength of O–H bonds intuitively, the Wiberg bond order in 1+0+0 (0.740) is smaller than that in a free water molecule (0.790), indicating that the sodium ion weakens the O–H bonds, in accordance with the results from NBO analysis.

**Figure 3:**
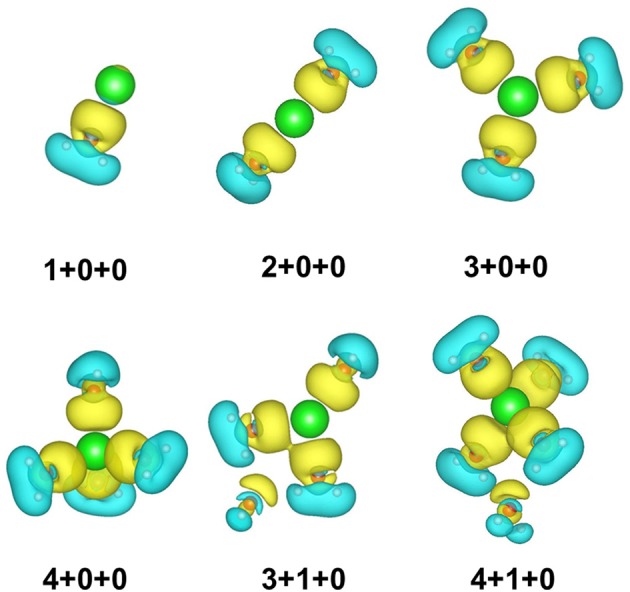
Charge density difference of six small hydrated sodium ion clusters. Yellow and blue spaces represent the electron accumulation and depletion regions, respectively.

To show the charge transfer of the whole clusters directly, the charge density difference of 1+0+0, 2+0+0, 3+0+0, 3+1+0, 4+0+0, and 4+1+0 is presented in Figure 3. The electrons from coordinated water molecules assemble at the location between sodium ions and water molecules near the side of oxygen atoms. The electron dissipation mostly happens near the hydrogen atoms, proving that the strengths of O–H bonds become weaker. In addition, the charge density difference of 3+1+0 and 4+1+0 show that the electrons also assemble at the location between the outer water molecules and the sodium ions, indicating that the ion–water interaction also reduces the strength of the O-H bonds in the outer water molecules.

## Vibrational Spectra

Vibrational spectrum is an intuitionistic method, providing deeper insight into structure differences and non-covalent interactions (Fan et al., 2019), especially O–H stretching vibration modes for hydrated sodium ion clusters. Therefore, the experimental Na^+^(H_2_O)_n_ isomers can be determined by comparing the simulated IR spectra and experimental spectra. Our discussion focuses on the high-frequency region (>3,200 cm^−1^) in IR spectra, which contains the O–H stretching vibration modes and can generally be measured in experiments (Ke et al., 2015).

**Figure 4:**
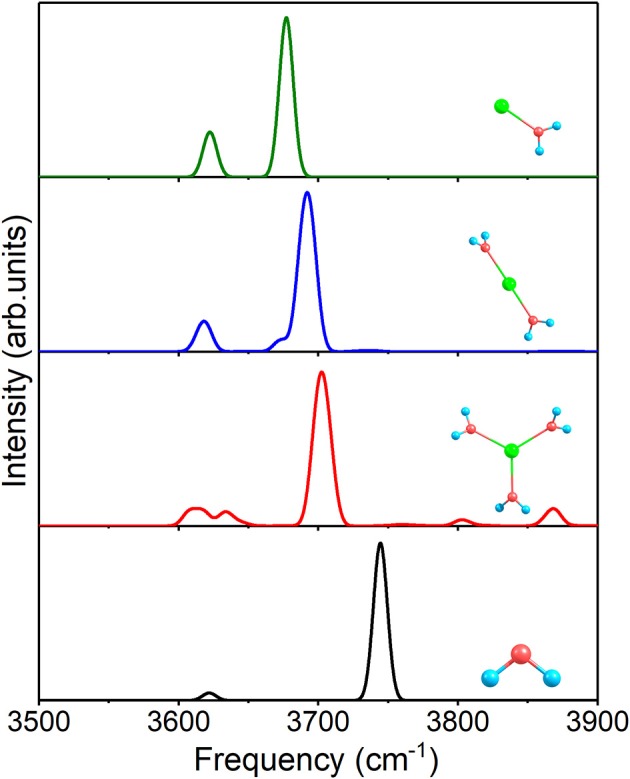
Anharmonic correctional IR spectra of 1+0+0, 2+0+0, 3+0+0 and a free water molecule calculated at MP2/aug-cc-pVDZ level of theory.

At first, Figure 4 shows the IR spectra of 1+0+0, 2+0+0, 3+0+0, and a free water molecule with anharmonic correction. The two O–H stretching vibrational modes are asymmetric (the higher peaks near 3,700 cm^−1^) and symmetric (the lower peaks near 3,620 cm^−1^) modes for each structure, and the other peaks are caused by anharmonic correction. Compared to the free water molecule, the asymmetric vibration modes of the three clusters possess redshifts, stemming from the ion–water interactions. The redshifts become smaller with the increasing of coordination number and $\bar{r}$*(Na–O)* in Table 2.

**Figure 5:**
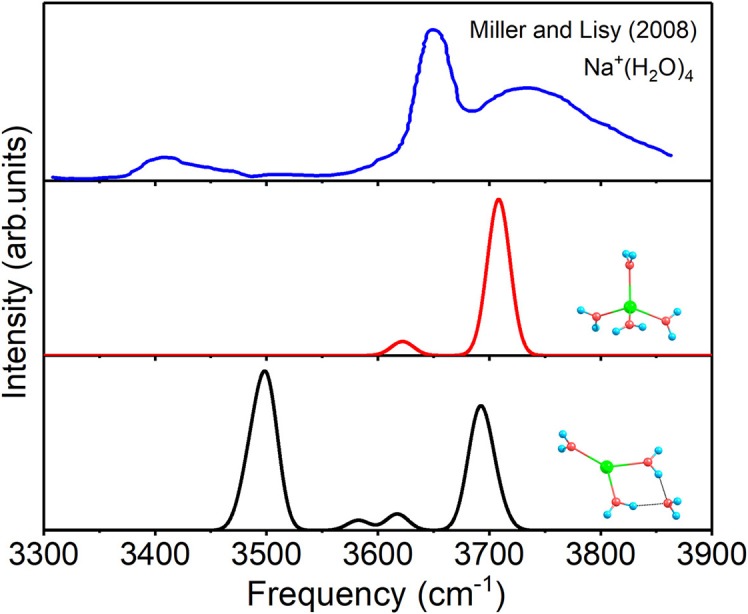
Anharmonic correctional IR spectra of 3+1+0, 4+0+0 calculated at MP2/aug-cc-pVDZ level of theory and experimental spectrum.

For *n* = 4, the spectra of 4+0+0 and 3+1+0, as well as the experimental spectrum of Na^+^(H_2_O)_4_ at 300 K (Miller and Lisy, 2008a) are given in Figure 5. The two modes of 4+0+0 reproduce the two outstanding peaks of experimental spectrum well. Moreover, the lower peak of the experimental spectrum confirms the small fraction of the existence of 3+1+0. Therefore, 4+0+0 dominates in the experiment at 300 K, and 3+1+0 is concomitant with 4+0+0, in accordance with the almost equal energies at 298 K in Table 1.

**Figure 6:**
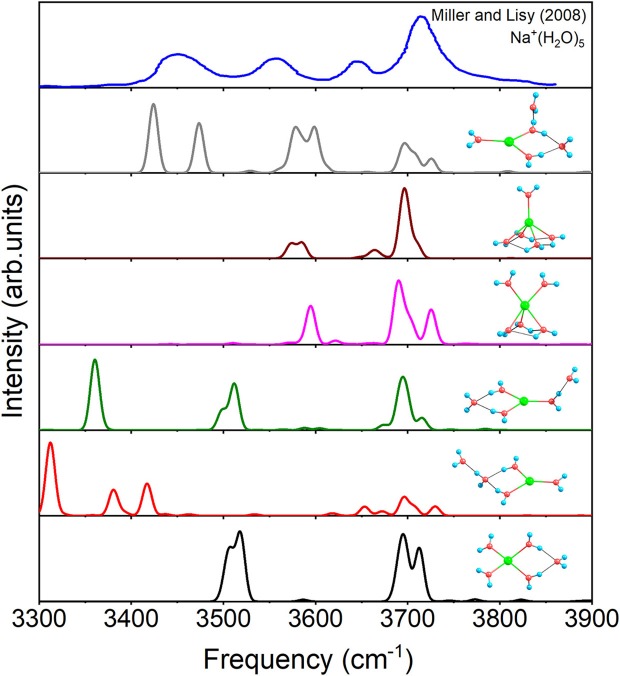
Anharmonic correctional IR spectra of 4+1+0, 3+1+1, 3+2+0(1), 5+0+0(1), 3+2+0(2), and 5+0+0(2) calculated at MP2/aug-cc-pVDZ level of theory and experimental spectrum.

Figure 6 shows the IR spectra of six isomers for *n* = 5 with anharmonic correction, as well as the experimental spectrum (Miller and Lisy, 2008a). Apparently, no single structure could reproduce the experimental spectrum well. Among, the vibrational modes of 3+1+1 are able to correspond three peaks of the experimental spectrum (Miller and Lisy, 2008a), hence 3+1+1 possesses the most possibility of existing in experiment. However, no mode in 3+1+1 could reproduce the experimental peak near 3,560 cm^−1^, while all the other five structures have vibrational modes near 3,560 cm^−1^. Combining with the relative energies in Figure 1, 4+1+0, the global minimum, could be the main contributor to the peak at 3,560 cm^−1^, in accordance with the conclusion in previous reports (Ke et al., 2015; Fifen and Agmon, 2016). Therefore, 4+1+0 and 3+1+1 are concomitant in experiments, which are the two lowest energetic structures at 298 K in Table 1.

**Figure 7:**
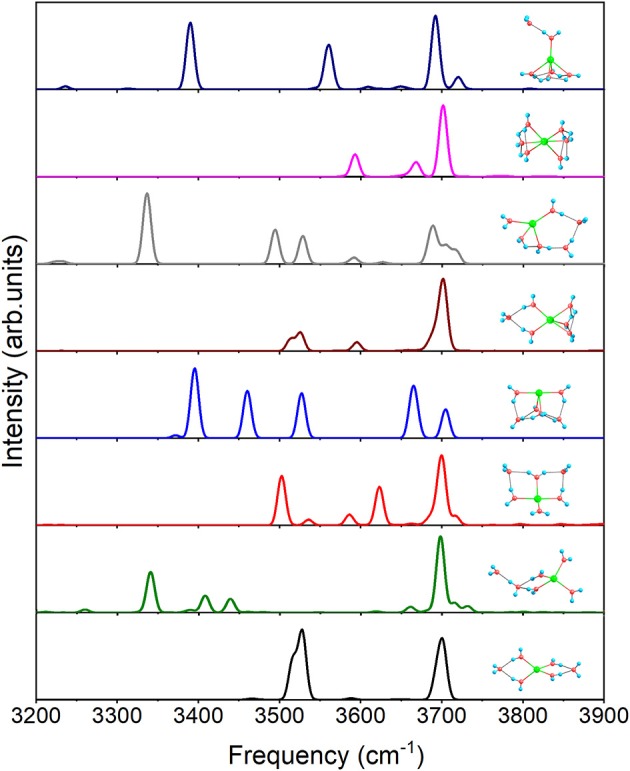
Anharmonic correctional IR spectra of eight isomers for *n* = 6 shown in Figure 1 calculated at MP2/aug-cc-pVDZ level of theory.

For *n* = 6, Figure 7 shows the IR spectra of all the eight isomers presented in Figure 1. Due to all the coordinated water molecules being equivalent to 4+2+0(1) in Figure 1, only two distinct peaks can be observed. Similar to 3+1+1, 4+1+1 possesses an obvious peak at 3340.5 cm^−1^ caused by O–H stretching of the water molecule in the second shell. 4+2+0(2) has no peak under 3,500 cm^−1^ because the hydrogen bonds are not strong enough to make the water molecules distinctly asymmetric, while the three feature peaks, 3371.5, 3395.4, and 3460.2 cm^−1^, of 4+2+0(3) are generated by the O–H stretching in the water cycle. Because of the hydrogen bonds between the outer molecule and coordinated water molecules in 5+1+0(1), the spectrum has two modes at 3513.3 and 3525.6 cm^−1^, which can't be found in 5+0+0(1). Due to no equivalent water molecule in 4+2+0(4), the O–H stretching vibration modes with different frequencies make the spectrum more complex than the others. 6+0+0 has a spectrum similar to that of 5+0+0(2) in Figure 6, corresponding to the similar water cycles in both structures. Unlike 5+0+0(2), 5+1+0(2) has a significant peak at 3390.0 cm^−1^ which is the O–H stretching mode of the proton-donating coordinated water molecule, indicating that the hydrogen bond between the outer water molecule and the coordinated water molecule is strong in 5+1+0(2).

## Conclusion

In this work, we investigate the geometries, energies, charges, and anharmonic vibrational properties of Na^+^(H_2_O)_n_ (*n* = 1–6). The CGA search and geometrical optimization for the cluster isomers provide accurate stable structures of Na^+^(H_2_O)_n_ (*n* = 1–6). At 0 K and 298 K, for *n* = 1–4, all the water molecules in global minima are coordination water molecules, surrounding the central sodium ions. Meanwhile, 4+1+0 and 4+2+0(1) are the global minima of *n* = 5 and 6, respectively. Thus, the coordination number of global minima of hydrated sodium ion clusters is 4 for *n* ≥ 4.

The non-covalent interactions, including ion–water interactions and hydrogen bonds, weaken the O–H bonds, resulting in longer bond lengths, lower bond orders, and redshifts of the O–H stretching mode in IR spectra. The simulated IR spectra with anharmonic correction can reproduce the experimental results well. The results show that 4+0+0 dominates for Na^+^(H_2_O)_4_, while 3+1+1 and 4+1+0 should be concomitant for Na^+^(H_2_O)_5_ in experiments. The present study executes a believable simulation of structures and vibrational spectra, and provides a comprehensive insight into the non-covalent interactions including ion–water interaction and hydrogen bonds of hydrated sodium ion clusters.

## Data Availability

The datasets generated for this study are available on request to the corresponding author.

## Author Contributions

PW and RS participated in the design and calculated the data of this study. PW, RS, and YS performed the statistical analysis. LT and XH improved the comprehensive genetic algorithm to adapt the system in this study. YS and JZ carried out the study and collected important background information. All authors have read and approved the content of the manuscript.

### Conflict of Interest Statement

The authors declare that the research was conducted in the absence of any commercial or financial relationships that could be construed as a potential conflict of interest.

## References

[B1] Arbman M. Siegbahn H. Pettersson L. Siegbahn P. (1985). Core electron-binding energies and auger-electron energies of solvated clusters - a computational study. Mol. Phys. 54, 1149–1160. 10.1080/00268978500100911

[B2] Bankura A. Carnevale V. Klein M. L. (2013). Hydration structure of salt solutions from ab initio molecular dynamics. J. Chem. Phys. 138:014501. 10.1063/1.4772761 23298049 PMC8487239

[B3] Bankura A. Carnevale V. Klein M. L. (2014). Hydration structure of Na^+^ and K^+^ fromab initiomolecular dynamics based on modern density functional theory. Mol. Phys. 112, 1448–1456. 10.1080/00268976.2014.905721

[B4] Barone V. (2005). Anharmonic vibrational properties by a fully automated second-order perturbative approach. J. Chem. Phys. 122:014108. 10.1063/1.1824881 15638643

[B5] Barone V. Bloino J. Guido C. A. Lipparini F. (2010). A fully automated implementation of VPT2 Infrared intensities. Chem. Phys. Lett. 496, 157–161. 10.1016/j.cplett.2010.07.012

[B6] Bauschlicher C. W. Langhoff S. R. Partridge H. Rice J. E. Komornicki A. (1991). A theoretical study of Na(H_2_O)^+^_*n*_ (*n* = 1–4). J. Chem. Phys. 95, 5142–5148. 10.1063/1.461682

[B7] Becke A. D. (1988). Density-functional exchange-energy approximation with coorect asymptotic-behavior. Phys. Rev. 38, 3098–3100. 10.1103/PhysRevA.38.3098 9900728

[B8] Biring S. K. Sharma R. Misra R. Chaudhury P. (2013). Structural and infrared spectroscopic aspects of ion-water clusters: a study based on a combined stochastic and quantum chemical approach. J. Cluster Sci. 24, 715–737. 10.1007/s10876-013-0565-4

[B9] Blades A. T. Jayaweera P. Ikonomou M. G. Kebarle P. (1990). Studies of alkaline earth and transition metal M^++^ gas phase ion chemistry. J. Chem. Phys. 92, 5900–5906. 10.1063/1.458360

[B10] Blochl P. E. (1994). Projector augmented-wave method. Phys. Rev. B 50, 17953–17979. 10.1103/PhysRevB.50.17953 9976227

[B11] Bush M. F. Saykally R. J. Williams E. R. (2008). Reactivity and infrared spectroscopy of gaseous hydrated trivalent metal ions. J. Am. Chem. Soc. 130, 9122–9128. 10.1021/ja801894d 18558673

[B12] Carpenter J. E. Weinhold F. (1988). Analysis of the geometry of the hydroxymethyl radical by the different hybrids for different spins natural bond orbital procedure. J. Mol. Struc-Theochem. 46, 41–62. 10.1016/0166-1280(88)80248-3

[B13] Carrillo-Tripp M. Saint-Martin H. Ortega-Blake I. (2003). A comparative study of the hydration of Na^+^ and K^+^ with refined polarizable model potentials. J. Chem. Phys. 118, 7062–7073. 10.1063/1.1559673

[B14] Cieplak P. Lybrand T. P. Kollman P. A. (1987). Calculation of free energy changes in ion–water clusters using nonadditive potentials and the Monte Carlo method. J. Chem. Phys. 86, 6393–6403. 10.1063/1.452428

[B15] Dang L. X. Rice J. E. Caldwell J. Kollman P. A. (1991). Ion solvation in polarizable water-molecular-dynamics simulations. J. Am. Chem. Soc. 113, 2481–2486. 10.1021/ja00007a021

[B16] Delley B. (2000). From molecules to solids with the DMol^3^ approach. J. Chem. Phys. 113, 7756–7764. 10.1063/1.1316015

[B17] Dinh P. M. Gao C. Z. Klüpfel P. Reinhard P. G. Suraud E. Vincendon M. (2014). A density functional theory study of Na(H_2_O)_n_: an example of the impact of self-interaction corrections. Eur. Phys. J. 68:239 10.1140/epjd/e2014-40816-1

[B18] Dudev T. Lim C. (2010). Factors Governing the Na^+^ vs K^+^ selectivity in sodium ion channels. J. Am. Chem. Soc. 132, 2321–2332. 10.1021/ja909280g 20108922

[B19] Dunning T. H. (1989). Gaussian-basis sets for use in correlated molecular calculations.1. the atoms boron through neon and hydrogen. J. Chem. Phys. 90, 1007–1023. 10.1063/1.456153

[B20] Dzidic I. Kebarle P. (1970). Hydration of the alkali ions in the gas phase. Enthalpies and entropies of reactions M^+^(H_2_O)_n−1_+H_2_O = M^+^(H_2_O)_n_. J. Phys. Chem. 74, 1466–1474. 10.1021/j100702a013

[B21] Fan J. Su Y. Zheng Z. Zhang Q. Zhao J. (2019). The pressure effects and vibrational properties of energetic material: Hexahydro-1, 3, 5-trinitro-1, 3, 5-triazine (α-RDX). J. Raman Spectrosc. 50, 889–898. 10.1002/jrs.5589

[B22] Feller D. Glendening E. D. Kendall R. A. Peterson K. A. (1994). An extended basis set ab initio study of Li^+^(H_2_O)_n_, n = 1–6. J. Chem. Phys. 100, 4981–4997. 10.1063/1.467217

[B23] Fifen J. J. Agmon N. (2016). Structure and spectroscopy of hydrated sodium ions at different temperatures and the cluster stability rules. J. Chem. Theory Comput. 12, 1656–1673. 10.1021/acs.jctc.6b00038 26913993

[B24] Frisch M. Trucks G. Schlegel H. Scuseria G. Robb M. Cheeseman J. (2013). Gaussian 09, Revision E.01. Wallingford, CT: Gaussian Inc.

[B25] Galib M. Baer M. D. Skinner L. B. Mundy C. J. Huthwelker T. Schenter J. K.. (2017). Revisiting the hydration structure of aqueous Na^+^. J. Chem. Phys. 146:084504. 10.1063/1.4975608 28249415

[B26] Glendening E. D. Feller D. (1995). Cation-water interactions: the M^+^(H_2_O)_n_ clusters for alkali metals M = Li, Na, K, Rb, and Cs. J. Phys. Chem. 99, 3060–3067. 10.1021/j100010a015

[B27] Hashimoto K. Morokuma K. (1994). Ab initio molecular orbital study of Na(H_2_O)_n_ (n = 1–6) clusters and their ions. Comparison of electronic structure of the Surface and Interior complexes. J. Am. Chem. Soc. 116, 11436–11443. 10.1021/ja00104a024

[B28] Hertel I. I. Huglin C. Nitsch C. Schulz C. P. (1991). Photoionization of Na(NH_3_)_n_ and Na(H_2_O)_n_ clusters: a step towards the liquid phase? Phys. Rev. Lett. 67, 1767–1770. 10.1103/PhysRevLett.67.1767 10044242

[B29] Huang Z. S. Miller R. E. (1989). High-resolution near-infrared spectroscopy of water dimer. J. Chem. Phys. 91, 6613–6631. 10.1063/1.457380

[B30] Jensen F. (1992). Structure and stability of complexes of glycine and glycine methyl analogs with H^+^, Li^+^, and Na^+^. J. Am. Chem. Soc. 114, 9533–9537. 10.1021/ja00050a036

[B31] Kamarchik E. Wang Y. Bowman J. M. (2011). Quantum vibrational analysis and infrared spectra of microhydrated sodium ions using an *ab initio* potential. J. Chem. Phys. 134:114311. 10.1063/1.3567186 21428623

[B32] Ke H. van der Linde C. Lisy J. M. (2015). Insights into the structures of the gas-phase hydrated cations M^+^(H_2_O)_n_Ar (M = Li, Na, K, Rb, and Cs; n = 3–5) using infrared photodissociation spectroscopy and thermodynamic analysis. J. Phys. Chem. A 119, 2037–2051. 10.1021/jp509694h 25651135

[B33] Kendall R. A. Dunning T. H. Harrison R. J. (1992). Electron-affinities of the 1st-row atoms revisited-systematic basis-sets and wave-functions. J. Chem. Phys. 96, 6796–6806. 10.1063/1.462569

[B34] Kim J. Lee S. Cho S. J. Mhin B. J. Kim K. S. (1995). Structures, energetics, and spectra of aqua-sodium(I): thermodynamic effects and nonadditive interactions. J. Chem. Phys. 102, 839–849. 10.1063/1.469199

[B35] Kozmutza C. Varga I. Udvardi L. (2003). Comparison of the extent of hydrogen bonding in H_2_O-H_2_O and H_2_O-CH_4_ systems. J. Mol. Struc-Theochem. 666, 95–97. 10.1016/j.theochem.2003.08.017

[B36] Kresse G. Furthmuller J. (1996). Efficient iterative schemes for ab initio total-energy calculations using a plane-wave basis set. Phys. Rev. B 54, 11169–11186. 10.1103/PhysRevB.54.11169 9984901

[B37] Lee C. T. Yang W. T. Parr R. G. (1988). Development of the colle-salvetti correlation-energy formula into a functional of the electron-density. Phys. Rev. B 37, 785–789. 10.1103/PhysRevB.37.785 9944570

[B38] Lee H. M. Tarakeshwar P. Park J. Kolaski M. R. Yoon Y. J. Yi H. B. (2004). Insights into the structures, energetics, and vibrations of monovalent cation-(Water)_1−6_ clusters. J. Phys. Chem. A 108, 2949–2958. 10.1021/jp0369241

[B39] Lev B. Roux B. Noskov S. Y. (2013). Relative free energies for hydration of monovalent ions from QM and QM/MM simulations. J. Chem. Theory Comput. 9, 4165–4175. 10.1021/ct400296w 26592407

[B40] Liu C. Min F. Liu L. Chen J. (2019). Hydration properties of alkali and alkaline earth metal ions in aqueous solution: a molecular dynamics study. Chem. Phys. Lett. 727, 31–37. 10.1016/j.cplett.2019.04.045

[B41] Liu Y. Zhao J. Li F. Chen Z. (2013). Appropriate description of intermolecular interactions in the methane hydrates: an assessment of DFT methods. J. Comput. Chem. 34, 121–131. 10.1002/jcc.23112 22949382

[B42] Lybrand T. P. Kollman P. A. (1985). Water–water and water–ion potential functions including terms for many body effects. J. Chem. Phys. 83:2923 10.1063/1.449246

[B43] Mancinelli R. Botti A. Bruni F. Ricci M. A. Soper A. K. (2007). Hydration of sodium, potassium, and chloride ions in solution and the concept of structure maker/breaker. J. Phys. Chem. 111, 13570–13577. 10.1021/jp075913v 17988114

[B44] Mano I. Driscoll M. (1999). DEG ENaC channels: a touchy superfamily that watches its salt. Bioessays 21, 568–578. 10.1002/(SICI)1521-1878(199907)21:7<568::AID-BIES5>3.0.CO;2-L 10472184

[B45] Megyes T. Balint S. Grosz T. Radnai T. Bako I. Sipos P. (2008). The structure of aqueous sodium hydroxide solutions: a combined solution X-ray diffraction and simulation study. J. Chem. Phys. 128:044501. 10.1063/1.2821956 18247963

[B46] Miller D. J. Lisy J. M. (2008a). Entropic effects on hydrated alkali-metal cations: infrared spectroscopy and ab initio calculations of M^+^(H2O)_(x = 2−5)_ cluster ions for M = Li, Na, K, and Cs. J. Am. Chem. Soc. 130, 15393–15404. 10.1021/ja803666m 18939842

[B47] Miller D. J. Lisy J. M. (2008b). Hydrated alkali-metal cations: infrared spectroscopy and ab initio calculations of M^+^(H2O)_(x = 2−5)_Ar cluster ions for M = Li, Na, K, and Cs. J. Am. Chem. Soc. 130, 15381–15392. 10.1021/ja803665q 18939843

[B48] Møller C. Plesset M. S. (1934). Note on an approximation treatment for many-electron systems. Phy. Rev. 46, 618–622. 10.1103/PhysRev.46.618

[B49] Muller-Dethlefs K. Hobza P. (2000). Noncovalent interactions: a challenge for experiment and theory. Chem. Rev. 100, 143–167. 10.1021/cr9900331 11749236

[B50] Neela Y. I. Mahadevi A. S. Sastry G. N. (2012). First principles study and database analyses of structural preferences for sodium ion (Na^+^) solvation and coordination. Struc. Chem. 24, 67–79. 10.1007/s11224-012-0032-0

[B51] Patwari G. N. Lisy J. M. (2003). Mimicking the solvation of aqueous Na^+^ in the gas phase. J. Chem. Phys. 118, 8555–8558. 10.1063/1.1574018

[B52] Payandeh J. Scheuer T. Zheng N. Catterall W. A. (2011). The crystal structure of a voltage-gated sodium channel. Nature 475, 353–358. 10.1038/nature10238 21743477 PMC3266868

[B53] Perdew J. P. Burke K. Ernzerhof M. (1996). Generalized gradient approximation made simple. Phys. Rev. Lett. 77, 3865–3868. 10.1103/PhysRevLett.77.3865 10062328

[B54] Perera L. Berkowitz M. L. (1991). Many-body effects in molecular dynamics simulations of Na+(H_2_O)_n_ and Cl^−^(H_2_O)_n_ clusters. J. Chem. Phys. 95:1954 10.1063/1.460992

[B55] Perez P. Lee W. K. Prohofsky E. W. (1983). Study of hydration of the Na^+^ ion using a polarizable water model. J. Chem. Phys. 79:388 10.1063/1.445534

[B56] Pohl H. R. Wheeler J. S. Murray H. E. (2013). Sodium and potassium in health and disease. Met. Ions. Life Sci. 13, 29–47. 10.1007/978-94-007-7500-8_2 24470088

[B57] Probst M. M. (1987). A study of the additivity of interactions in cation-water systems. Chem. Phys. Lett. 137, 229–233. 10.1016/0009-2614(87)80210-5

[B58] Ramaniah L. M. Bernasconi M. Parrinello M. (1998). Density-functional study of hydration of sodium in water clusters. J. Chem. Phys. 109, 6839–6843. 10.1063/1.477250

[B59] Rao J. S. Dinadayalane T. Leszczynski J. Sastry G. N. (2008). Comprehensive study on the solvation of mono-and divalent metal cations: Li^+^, Na^+^, K^+^, Be^2+^, Mg^2+^ and Ca^2+^. J. Phys. Chem. 112, 12944–12953. 10.1021/jp8032325 18834092

[B60] Reed A. E. Curtiss L. A. Weinhold F. (1988). Intermolecular interactions from a natural bond orbital, donor-acceptor viewpoint. Chem. Rev. 88, 899–926. 10.1021/cr00088a005

[B61] Reimers J. R. Watts R. O. Klein M. L. (1982). Intermolecular potential functions and the properties of water. Chem. Phys. 64, 95–114. 10.1016/0301-0104(82)85006-4

[B62] Schulz C. P. Haugstatter R. Tittes H. U. Hertel I. I. (1986). Free sodium-water clusters. Phys. Rev. Lett. 57, 1703–1706. 10.1103/PhysRevLett.57.1703 10033523

[B63] Schulz C. P. Haugstatter R. Tittes H. U. Hertel I. V. (1988). Free sodium-water clusters-photoionisation studies in a pulsed molecular-beam source. Z. Phys. 10, 279–290. 10.1007/BF01384862

[B64] Shi R. Huang X. Su Y. Si H.-G. Lo S.-D. Tang L.. (2017). Which density functional should be used to describe protonated water clusters? J. Phys. Chem. 121, 3117–3127. 10.1021/acs.jpca.7b00058 28383918

[B65] Shi R. L. Wang P. J. Tang L. L. Huang X. M. Chen Y. G. Su Y.. (2018). Structures and Spectroscopic Properties of F^−^(H_2_O)_n_ with n = 1–10 Clusters from a Global Search Based On Density Functional Theory. J. Phys. Chem. 122, 3413–3422. 10.1021/acs.jpca.7b08872 29546760

[B66] Snyder P. M. (2002). The epithelial Na^+^ channel: cell surface insertion and retrieval in Na^+^ homeostasis and hypertension. Endocr. Rev. 23, 258–275. 10.1210/edrv.23.2.0458 11943747

[B67] Soniat M. Rogers D. M. Rempe S. B. (2015). Dispersion- and exchange-corrected density functional theory for sodium ion hydration. J. Chem. Theory Comput. 11, 2958–2967. 10.1021/acs.jctc.5b00357 26575733

[B68] Tang I. N. Castleman A. W. (1972). Mass-spectrometric study of gas-phase hydration of monovalent lead ion. J. Chem. Phys. 57, 3638–3644. 10.1063/1.1678820

[B69] Vaden T. D. Forinash B. Lisy J. M. (2002). Rotational structure in the asymmetric OH stretch of Cs^+^(H_2_O)Ar. J. Chem. Phys. 117, 4628–4631. 10.1063/1.1503310

[B70] Vaden T. D. Lisy J. M. Carnegie P. D. Pillai E. D. Duncan M. A. (2006). Infrared spectroscopy of the Li^+^(H_2_O)Ar complex: the role of internal energy and its dependence on ion preparation. Phys. Chem. Chem. Phys. 8, 3078–3082. 10.1039/b605442k 16804607

[B71] Vaden T. D. Weinheimer C. J. Lisy J. M. (2004). Evaporatively cooled M^+^(H_2_O)Ar cluster ions: infrared spectroscopy and internal energy simulations. J. Chem. Phys. 121, 3102–3107. 10.1063/1.1774157 15291620

[B72] Wiberg K. B. (1968). Application of pople-santry-segal cndo method to cyclopropylcarbinyl and cyclobutyl cation and to bicyclobutane. Tetrahedron 24, 1083–1096. 10.1016/0040-4020(68)88057-3

[B73] Zhao J. Shi R. Sai L. Huang X. Su Y. (2016). Comprehensive genetic algorithm for ab initio global optimisation of clusters. Mol. Simulat. 42, 809–819. 10.1080/08927022.2015.1121386

