# Supplementary material for: Hydrated Sodium Ion Clusters [Na+(H2O)n (n = 1–6)]: An ab initio Study on Structures and Non-covalent Interaction
Source: Front Chem. 2019 Sep 12;7:624. doi: 10.3389/fchem.2019.00624 (PMC6751288; doi:10.3389/fchem.2019.00624)
Supplement: Supplementary file 1 [file Table_1.DOCX]

**Table S1 | Relative energies (in units of kcal/mol) of Na^+^(H_2_O)_n_ (*n* = 1–6) at 0 K calculated at MP2/aug-cc-pVQZ and BLYP/DNP levels without ZPE, respectively.**

|  | | MP2/aug-cc-pVQZ | BLYP/DNP |
| --- | --- | --- | --- |
| 1+0+0 | 0 | | 0 |
| 2+0+0 | 0 | | 0 |
| 3+0+0 | 0 | | 0 |
| 3+1+0 | 0 | | 0 |
| 4+0+0 | 0.97 | | 0.01 |
| 4+1+0 | 0 | | 0 |
| 3+1+1 | 0.97 | | 1.04 |
| 3+2+0(1) | 1.04 | | 1.04 |
| 3+2+0(2) | 2.29 | | 2.52 |
| 5+0+0(2) | 2.30 | | 3.83 |
| 5+0+0(1) | 2.67 | | 4.05 |
| 4+2+0(3) | 0 | | 0 |
| 4+2+0(1) | 0.14 | | –0.88 |
| 4+2+0(2) | 1.61 | | 0.71 |
| 4+1+1 | 2.28 | | 1.00 |
| 5+1+0(1) | 2.39 | | 2.77 |
| 5+1+0(2) | 3.01 | | 4.50 |
| 4+2+0(4) | 3.44 | | 2.40 |
| 6+0+0 | 4.31 | | 5.56 |

**Table S2 | Distances between sodium ions and oxygen atoms (*r(Na–O)*), O–H bond lengths (*r(O–H)*) and H–O–H bond angles (*∠(H–O–H)*) of 2+0+0 optimized at MP2/aug-cc-pVDZ and MP2/aug-cc-pVTZ levels, respectively.**

|  | *r(Na–O)*/Å | *r(O–H)*/Å | *∠(H–O–H)*/° |
| --- | --- | --- | --- |
| MP2/aug-cc-pVDZ | 2.302 | 0.968 | 104.0 |
| MP2/aug-cc-pVTZ | 2.307 | 0.964 | 104.2 |
